# The Association Between Body Mass Index and the Prognosis and Postoperative Complications of Hepatocellular Carcinoma: A Meta-Analysis

**DOI:** 10.1097/MD.0000000000001269

**Published:** 2015-08-07

**Authors:** Xiaoxiang Rong, Fang Wei, Qian Geng, Jian Ruan, Hongfen shen, Aimin Li, Rongcheng Luo

**Affiliations:** From the Department of Oncology, Nanfang Hospital (XR); Department of Oncology, Traditional Chinese Medicine-Integrated Hospital (XR, QG, JR, AL, RL); Cancer Research Institute, Southern Medical University, Guangzhou (FW, HS); Department of Oncology, Changzhou No.2 People's Hospital, The Affiliated Hospital of Nanjing Medical University, Changzhou (QG); and Guangzhou Digestive Disease Center, Guangzhou First People's Hospital, Guangzhou Medical University, Guangzhou, Guangdong Province, China (FW).

## Abstract

Previous studies have reported the association between excess body mass index (BMI) and increased risk of hepatocellular carcinoma (HCC). However, whether BMI is associated with the prognosis and postoperative complications of HCC is still not clear.

We searched PubMed and Embase for relevant studies published until the date of August 30, 2014. Additional studies were manually identified by searching reference lists of retrieved articles. Pooled hazard ratios (HRs) with 95% confidence intervals (95% CIs) for overall survival (OS), disease-free survival (DFS), and risk ratios (RRs) with 95% CIs for postoperative complications were calculated using random effects or fixed effects models according to heterogeneities between studies.

A total of 14 studies were included in the present meta-analysis. The pooled results showed that excess BMI was not significantly associated with improved OS (HR = 0.94; 95% CI: 0.74–1.19, *P* = 0.588) or DFS (HR = 0.93; 95% CI: 0.79–1.10, *P* = 0.382). In addition, higher BMI was not associated with increased rate of a number of complications including ascites (RR = 1.25, 95% CI: 0.94–1.65, *P* = 0.119), bile leaks (RR = 1.22, 95% CI: 0.81–1.83, *P* = 0.345), and 30-day mortality (RR = 1.05, 95% CI: 0.57–1.96, *P* = 0.871). However, HCC patients with higher BMI had increased incidence of wound infections (RR = 2.17, 95% CI: 1.28–3.68, *P* = 0.004).

BMI was not an independent prognostic factor for the evaluation of the prognosis in HCC patients, and it was not associated with postoperative complications except for wound infections that as significantly associated with higher BMI scores.

## INTRODUCTION

Hepatocellular carcinoma (HCC) is one of the most common human cancers, especially in Asian countries. Despite significant progress has been made in medical research and surgical techniques, HCC is still a fatal disease with poor prognosis. Approximately 500,000 new cases of HCC is reported annually worldwide,^1^ and a nearly equivalent number of patients die from this disease.^2^

It has been reported that a number of lifestyle factors such as physical activities, diet, and overweight were associated with various human cancers.^3^ For example, previous studies provided evidence showing that body mass index (BMI) was associated with the risk of HCC.^4,5^ The recent meta-analysis including 21 prospective studies has shown that excess BMI significantly increased the risk of HCC.^5^ A number of studies have investigated the association between BMI and the prognosis and postoperative complications of HCC. Three studies demonstrated that HCC patients with higher BMI exhibited significantly better prognosis than HCC patients with lower BMI after hepatic resection surgery.^6–8^ However, no significant differences in the prognosis were detected between the 2 groups with different levels of BMI in other studies.^9–14^ In addition, 2 studies reported that obesity had negative impacts on postoperative complications in HCC patients after hepatectomy,^8,15^ whereas conflicting results have been reported by other studies.^6,7,16–18^ Therefore, the present study aimed to investigate the association between BMI and the prognosis of HCC as well as postoperative complications in HCC patients after hepatectomy.

## MATERIALS AND METHODS

### Search Strategy and Selection Criteria of Literatures

Literatures published until the date of August 30, 2014 were searched in the Embase and PubMed databases by 2 independent investigators using the keywords “body mass index,” “BMI,” “overweight,” or “obesity” in combination with “hepatocellular carcinoma,” “HCC,” or “liver cancer.” In addition, the references of each literature were also examined to identify appropriate articles. Duplicated literatures were finally removed. Because the data included in our study were extracted from published literatures, ethical approval from ethics committees was not needed.

Studies were included in the present meta-analysis according to the following criteria: HCC patients underwent liver resection; BMI as an exposure interest; available data for estimating parameters; and published in English.

### Data Extraction

Two investigators independently extracted the following data from each study: first author's name, publication year, study location, sample size, BMI categories, covariates adjusted for in the analysis, hazard ratio (HR) with 95% confidence interval (95% CI) for overall survival (OS), time to progression (TTP), and relative risk ratios (RRs) with 95% CI for postoperative complications.

### Statistical Analyses

Meta-analyses were conducted using STATA 12.0 according to the Cochrane Handbook for Systematic Reviews of Interventions.^19^ For the time-to-event variables, HRs with 95% CI were directly extracted from each study. When the association between BMI and HRs of survival was not reported, HRs were calculated according to the methods described by Parmar et al and Tierney et al.^20,21^ RRs with 95% CI were calculated for analyzing postoperative complications. Heterogeneity between studies was evaluated with Cocharan Q test as well as I^2^ index.^19^ A random effects model was used for studies with heterogeneity. Otherwise, a fixed effects model was used when heterogeneity was not identified.

To identify potential sources of heterogeneity between studies, stratified analyses were conducted according different publication years, geographic locations, BMI cutoffs, case size, duration of follow-up, and methods of data acquired, respectively. In addition, to evaluate the sensitivity of the meta-analysis, each article was omitted from the analysis and the results were compared with the overall results. The Begg funnel plot and Egger test were used to estimate potential publication bias. The significance of the pooled HR or RR was determined using the Z-test and a *P* value less than 0.05 was considered as statistically significant.

## RESULTS

### Characteristics of the Included Studies

**FIGURE 1:**
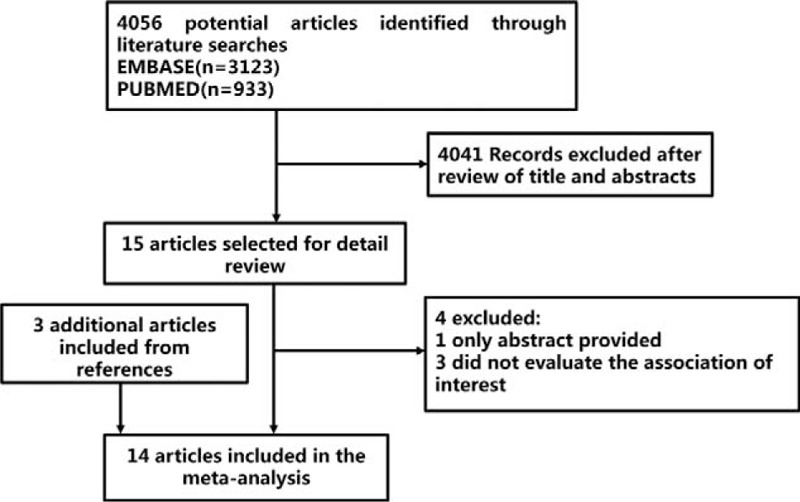
The flowchart showing the selection and inclusion criteria of published literatures for the present meta-analysis.

A flow chart showing the selection of qualified literatures for the present meta-analysis was shown in Figure 1. Finally, 14 studies that met our selection criteria were included in the present meta-analysis.^6–18,22^ The detailed characteristics of the 14 studies, including the first author's names, publication years, the countries where the studies performed, sample size, virology test results, and BMI, were described in Table 1. Of the 14 studies, 8, 2, 2, 1, and 1 studies were conducted in Japan, the United States, China, Italy, and France, respectively. All articles were published in English and 10 articles (71%) were published after 2010. Among the studies, only 1 study was about intraabdominal cancer.^22^ Due to distinct cutoff values of BMI were used in different studies, pooled estimates of HR or RR were calculated with the comparison of the highest BMI group and the lowest group for consistency.

**TABLE 1:**
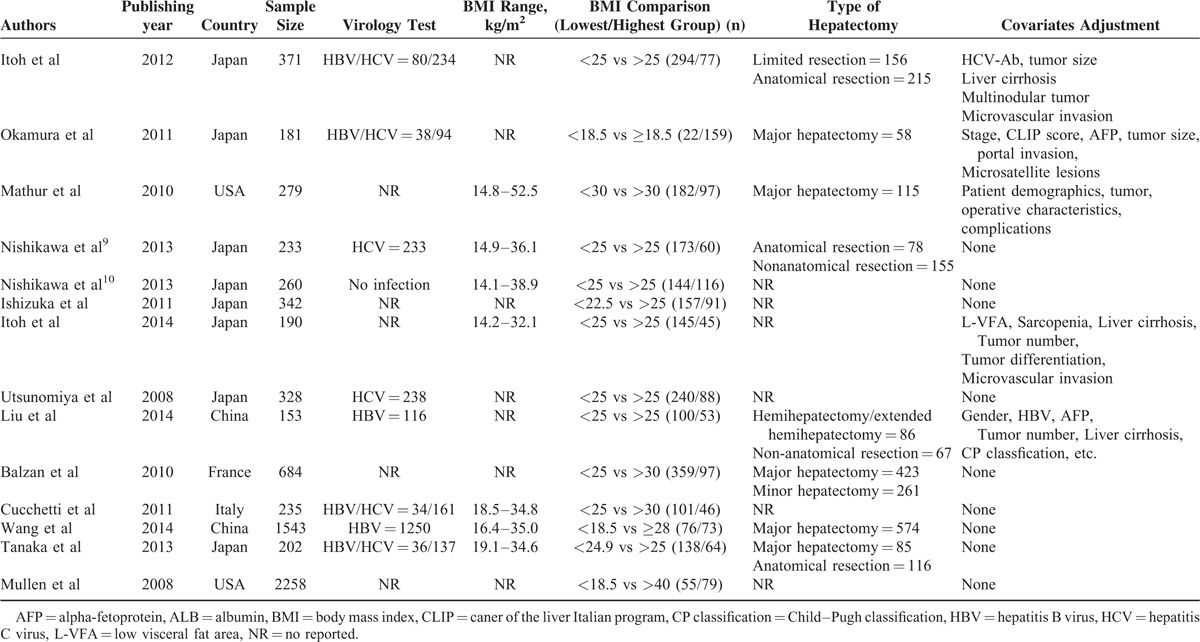
Characteristics of the 14 Published Studies Included in the Present Meta-Analysis

### Meta-Analysis of the Association Between BMI and the Overall Survival and Disease-Free Survival

**FIGURE 2:**
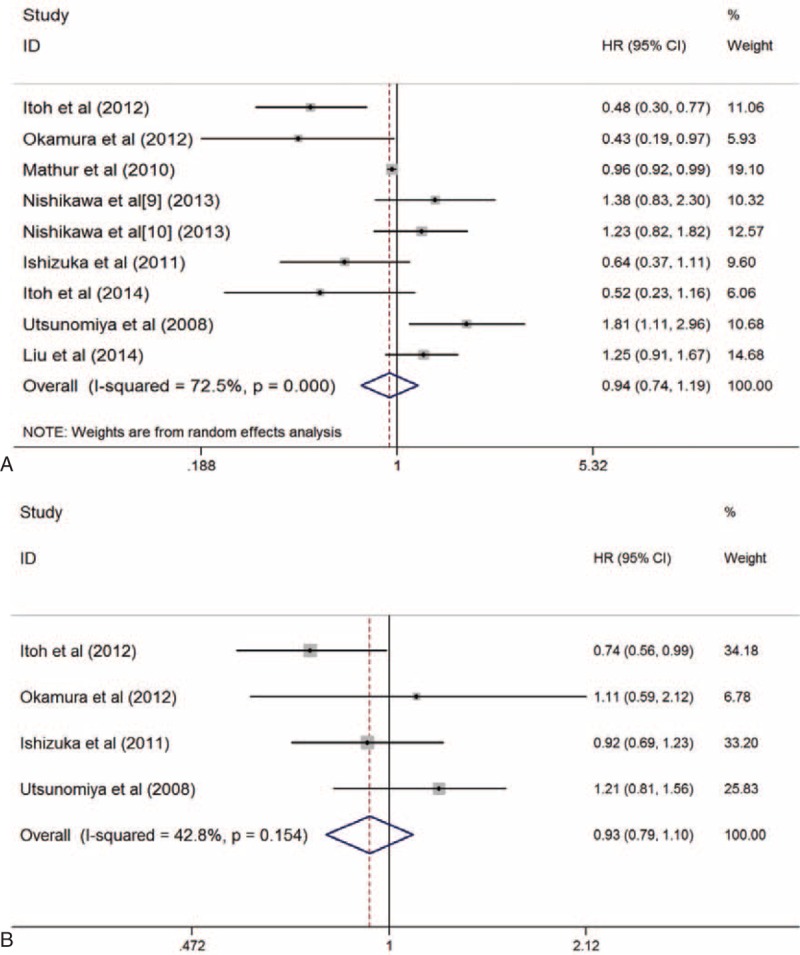
The Forest plots of HR for the OS (A) and DFS (B) of patients with the highest and lowest BMI categories, respectively. BMI = body mass index, DFS = disease-free survival, HR = hazard ratio, OS = overall survival.

Of the 14 included studies, 9 studies^6–14^ presented available data of the HR for OS. Among them, 3 studies^6–8^ demonstrated that patients with higher BMI had better prognosis, whereas no significant difference in OS was identified between patients with different BMI in the other 6 studies. Significant heterogeneity was observed between these studies; therefore, the random-effects mode was used in follow-up analyses. The pooled HR for OS was 0.94 (95% CI: 0.74–1.19; *P* = 0.588; I^2^ = 72.5%, *P* = 0.000) (Figure 2A). The Egger test (*P* = 0.839) and Begg test (*P* = 0.251) showed no publication bias.

Four studies^6,7,11,13^ were included in disease-free survival (DFS) analysis. The pooled HR for DFS was 0.93 (95% CI: 0.79–1.10; *P* = 0.382; I^2^ = 42.8%, *P* = 0.154) (Figure 2B), suggesting that BMI was not an appropriate factor for the evaluation of prognosis. The Egger test (*P* = 0.577) and Begg test (*P* = 0.308) showed no publication bias.

### Meta-Analysis of the Association Between BMI and Postoperative Complications

**FIGURE 3:**
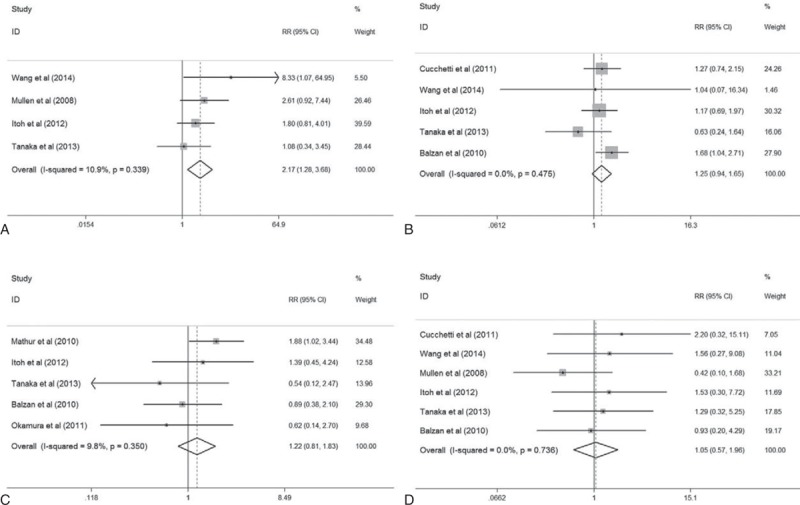
The Forest plots of RR for the postoperative complications including wound infection (A), ascites (B), bile leaks (C), and 30-day mortality (D) of patients with the highest and lowest BMI categories, respectively. BMI = body mass index, RR = risk ratio

To evaluate the association between BMI and postoperative complications, 8 studies that have reported postoperative complications were analyzed.^6–8,15–18,22^ As shown in Figure 3, a significant association between higher BMI and increased complications of wound infection was identified (RR = 2.17, 95% CI: 1.28–3.68, *P* = 0.004) (Figure 3A). No significant association was identified between BMI and the incidence of ascites (RR = 1.25, 95% CI: 0.94–1.65, *P* = 0.119) (Figure 3B), bile leaks (RR = 1.22, 95% CI: 0.81–1.83, *P* = 0.345) (Figure 3C), and 30-day mortality (RR = 1.05, 95% CI: 0.57–1.96, *P* = 0.871) (Figure 3D). For all pooled estimates mentioned above, no significant heterogeneity was detected.

### Subgroup Analyses and Sensitivity Analyses

To explore potential sources of heterogeneity, we conducted subgroup analyses according to different publication years, geographic locations, BMI cutoff values, case size, duration of follow-up, and methods of data acquired (Table 2). The heterogeneity was significantly reduced after the studies published before 2012 were excluded (HR = 1.16, 95% CI: 0.88–1.53; *P* = 0.283; I^2^ = 32.9%, *P* = 0.215), suggesting that publication years are one of the sources of inconsistency between studies. According to the cutoff value of BMI, analysis was performed in the subgroup of 6 studies with the same BMI cutoff value 25. No statistically significance between BMI and OS was found in the BMI cutoff value 25 subgroup with significant heterogeneity (*P* for heterogeneity = 0.000). When the studies were grouped by the other factors such as country, case size, duration of follow-up, and method of data acquired, the heterogeneity still existed.

**TABLE 2:**
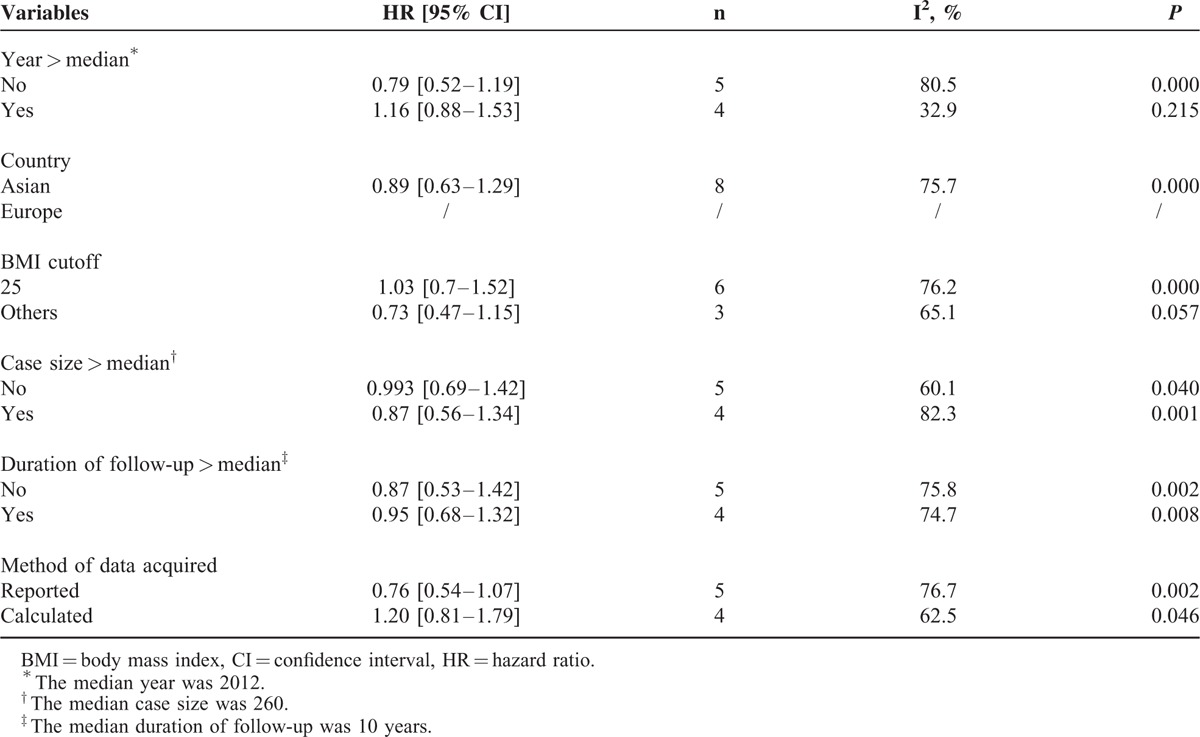
The Results of Subgroup Analyses on Overall Survival (OS)

Given the detection of heterogeneity between these studies, we conducted sensitivity analyses by omitting each article in turn, and comparing the results among them. We found that the overall results were not influenced by removal of any study, suggesting the high stability of our meta-analysis results (Table 3).

**TABLE 3:**
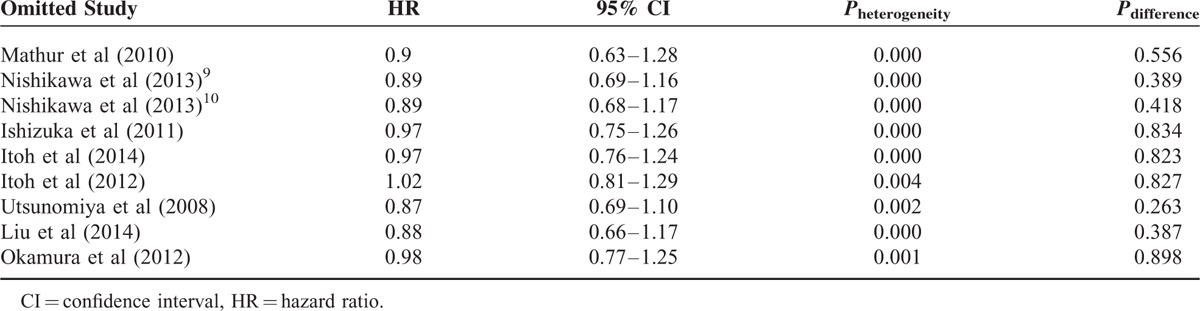
The Results of Sensitivity Analyses on Overall Survival (OS)

## DISCUSSION

It has been reported that BMI was associated with the risk of HCC.^4,5^ However, it remains unclear whether BMI is associated with the prognosis of HCC patients and whether it is a reliable prognostic factor for evaluating the survival of HCC patients after hepatotectomy. In addition, whether higher BMI was associated with increased incidence of postoperative complication is still debated. Recently, a number of studies have evaluated the relationship between BMI and the prognosis and postoperative complications; however, the results of these studies are conflicting.^6–18,22^ To the best of our knowledge, this is the first meta-analysis systematically evaluating the association between BMI and the prognosis and postoperative complications in HCC patients based on previous studies.

Notably, it has been reported that excess BMI was associated with distinct outcomes of different cancers. For example, excess BMI significantly increased the risk of renal cell carcinoma (RCC), but higher BMI was associated with better prognosis in RCC patients,^23,24^ similar findings were found in oesophageal cancer.^25^ In contrast, pancreatic cancer patients with excess BMI had poorer prognosis than those with normal BMI after pancreatic resection surgery.^26^ In the present study, our results suggest that BMI was not a significant prognostic factor for HCC. No significant differences in the OS or DFS rates were detected between the 2 groups with different BMI scores. However, the underlying mechanisms of the heterogeneous relationship between BMI and the risk and prognosis of various cancers has rarely been elucidated and needed to be further studied. Given that significant heterogeneities are found in the present meta-analysis, stratified and sensitivity analyses were performed to explore the potential sources of these heterogeneities. Our results suggest that the publishing year was one of the major source of heterogeneities. Although heterogeneities still existed when the included studies were stratified according to other factors such as BMI cutoff values. Therefore, the stratified analysis failed. Considering the existence of heterogeneity, our results need to be further confirmed by large-scale clinical trials.

In the present study, HCC patients with excess BMI had increased incidence of wound infections. However, no significant differences in the other postoperative complications such as ascites, bile leaks, and 30-day mortality were observed between the 2 groups with different BMI scores. Our findings were consistent with a number of previous studies. In a cohort study including 6336 patients, obese patients had similar postoperative complications after general surgeries as patients with normal BMI except for wound infections that were significantly associated with patients with higher BMI.^26^ Similarly, Hawn et al^27^ reported that obese patients experienced significantly longer operative time, but no significant differences in postoperative complications except for wound infections were found between patients with distinct BMI scores.^27^ Wound infections occurred more frequently in patients with excess BMI. The relative higher rate of wound infections in patients with excess BMI could be explained by the following reasons. First, adipose tissue usually has lower blood perfusion and oxygen tension than other tissues, which may increase the risk of wound infection. Second, obese patients usually have more difficulties in exposure during surgery, which typically requires larger surgical incisions. Third, immune deficiency is more common in obese patients.^28^ Finally, obese patients have higher rate of diabetes mellitus, which delays the recovery of wound. However, the sample size in these studies on the evaluation of the association of BMI and postoperative complication was relatively small; therefore, the results of these studies and our study should be further confirmed based on larger sample size.

A number of limitations that may affect the interpretation of the results in the present should be discussed. First, significant heterogeneities existed in our study. Although the heterogeneity became nonsignificant after the studies published before 2012 were excluded, the heterogeneity was not totally removed through stratified analyses. Second, the included studies may have failed to control for known or unknown covariates. For instance, only 2 studies had considered hepatitis infections in HCC patients.^6,14^ Alcohol intake was not adjusted in any of these studies, and only 3 studies have adjusted for liver cirrhosis.^6,12,14^ Lack of adjustment for these factors may limit the ability of the present meta-analysis for evaluating the association between BMI and HCC risk and prognosis. In addition, we were not able to perform a more detailed subgroup analysis based on the disease status of HCC patients, because the published studies included in our meta-analysis did not provide relative results.

In summary, the present meta-analysis suggests that higher BMI was not associated with the prognosis and postoperative complications except for wound infections in HCC patients. HCC patients with higher BMI scores exhibited higher rate of wound infections.
